# A coupled enzyme assay for detection of selenium-binding protein 1 (SELENBP1) methanethiol oxidase (MTO) activity in mature enterocytes

**DOI:** 10.1016/j.redox.2021.101972

**Published:** 2021-04-15

**Authors:** Thilo Magnus Philipp, Andreas Will, Hannes Richter, Patrick Rainer Winterhalter, Georg Pohnert, Holger Steinbrenner, Lars-Oliver Klotz

**Affiliations:** a Institute of Nutritional Sciences, Nutrigenomics Section, Friedrich Schiller University Jena, Jena, Germany; b Institute for Inorganic and Analytical Chemistry, Friedrich Schiller University Jena, Jena, Germany; c Department of Cardiac Surgery, Martin Luther University Halle-Wittenberg, Halle, Germany

**Keywords:** Selenium-binding protein 1, Methanethiol oxidase, Caco-2 cells, Hydrogen sulfide, Hydrogen peroxide

## Abstract

Methanethiol, a gas with the characteristic smell of rotten cabbage, is a product of microbial methionine degradation. In the human body, methanethiol originates primarily from bacteria residing in the lumen of the large intestine. Selenium-binding protein 1 (SELENBP1), a marker protein of mature enterocytes, has recently been identified as a methanethiol oxidase (MTO). It catalyzes the conversion of methanethiol to hydrogen sulfide (H_2_S), hydrogen peroxide (H_2_O_2_) and formaldehyde. Here, human Caco-2 intestinal epithelial cells were subjected to enterocyte-like differentiation, followed by analysis of SELENBP1 levels and MTO activity. To that end, we established a novel coupled assay to assess MTO activity mimicking the proximity of microbiome and intestinal epithelial cells *in vivo*. The assay is based on *in situ*-generation of methanethiol as catalyzed by a bacterial recombinant l-methionine gamma-lyase (MGL), followed by detection of H_2_S and H_2_O_2_. Applying this assay, we verified the loss and impairment of MTO function in SELENBP1 variants (His329Tyr; Gly225Trp) previously identified in individuals with familial extraoral halitosis. MTO activity was strongly enhanced in Caco-2 cells upon enterocyte differentiation, in parallel with increased SELENBP1 levels. This suggests that mature enterocytes located at the tip of colonic crypts are capable of eliminating microbiome-derived methanethiol.

## Highlights

• An MTO assay is presented, imitating intestinal methanethiol generation/elimination.
• Using this assay, known human SELENBP1 mutants were found to be MTO-deficient.
• SELENBP1/MTO activity increases during enterocytic differentiation of Caco-2 cells.
• SELENBP1 provides a means of coping with microbiome-derived methanethiol.
• SELENBP1 is a source of H_2_S and H_2_O_2_ in cells exposed to methanethiol.

## Graphical abstract

**Figure:**
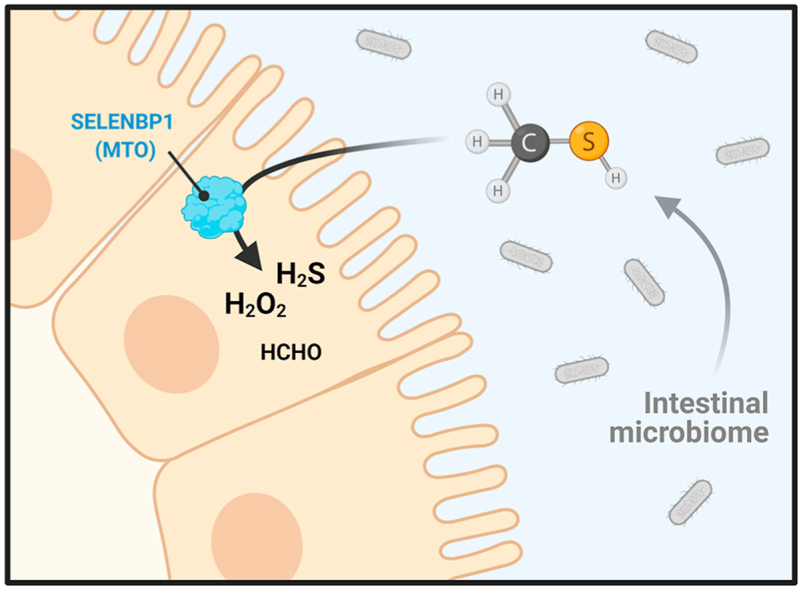

## 1 Introduction

Selenium-binding protein 1 (SELENBP1) was previously hypothesized to act as a tumor suppressor [1], and to be involved in intracellular protein degradation and transport [2,3]. It is not a selenoprotein but was proposed to bind selenium in the form of selenite [4], although it has not been fully elucidated yet how selenium affects its biological activities. Recently, SELENBP1 was identified as a methanethiol oxidase (MTO), catalyzing the conversion of methanethiol (H_3_C-SH) to hydrogen sulfide (H_2_S), hydrogen peroxide (H_2_O_2_) and formaldehyde (HCHO) [5]. Methanethiol is a volatile and toxic gas with the characteristic smell of rotten cabbage, contributing to the manifestation of extraoral halitosis in humans [5]. Although mitochondrial degradation of methionine was reported as a cellular source of methanethiol in mammals [6], this pathway appears to be of some significance only under conditions of methionine excess and deficiencies in the transsulfuration pathway [7,8] (for a recent review, see Ref. [9]). Thus, in humans, the vast majority of methanethiol is produced by the intestinal microbiome. This is supported by reports on lowered methanethiol production in humans undergoing antibiotic treatment [5]. Bacterial generation of methanethiol results from their degradation of methionine as catalyzed by methionine γ-lyase (MGL), an enzyme that humans are deficient in [10].

*SELENBP1* is ubiquitously expressed, but its highest mRNA and protein levels were found in gastrointestinal tissues, liver and lung, as suggested by Pol et al. [5] and data available through the Human Protein Atlas (version 20.0, https://www.proteinatlas.org/ENSG00000143416-SELENBP1/tissue) [11]. Interestingly, *SELENBP1* expression was described to be strongly induced in the course of cell differentiation not only in adipocytes [12] but also in intestinal epithelial cells [13,14], in the latter case resulting in an increase in SELENBP1 protein levels along the colonic crypt-luminal axis [13]. This would put cellular SELENBP1 in close proximity to the gut microbiome, the major (extracellular) source of its substrate, methanethiol. Conversion of methanethiol to H_2_S by colonic mucosa was reported already 20 years ago, but the enzyme in charge was unknown at the time [15].

We hypothesized that an increase in SELENBP1 levels during intestinal differentiation comes with elevated MTO activity, and we developed a simple reliable enzymatic assay that imitates the *in vivo* situation (bacterial generation of methanethiol and mammalian degradation of methanethiol) to test this hypothesis. Moreover, we used this assay to evaluate the impact of amino acid exchanges in SELENBP1 on its MTO activity.

## 2 Materials and methods

### 2.1 Cultivation and differentiation of Caco-2 cells

Caco-2 human colon adenocarcinoma cells (German Collection of Microorganisms and Cell Cultures, DSMZ, Braunschweig, Germany) were a kind gift of Dr. R. Schins (Leibniz Research Institute for Environmental Medicine, IUF, Düsseldorf, Germany). Cells were cultivated as previously described [16] in Minimum Essential Medium Eagle (MEM, #M4655; Sigma-Aldrich, Deisenhofen, Germany) supplemented with 20% (v/v) fetal bovine serum (FBS, #S0615; Sigma-Aldrich), 1% non-essential amino acids (#M7145; Sigma-Aldrich) and 1% penicillin/streptomycin (#P4333; Sigma-Aldrich) at 37 °C in a humidified atmosphere with 5% (v/v) CO_2_. For the experiments, cells (6 × 10^4^/cm^2^) were seeded in 10 cm (diameter)-dishes and grown either to confluence at day 0 (undifferentiated cells) or until day 14 after reaching confluence (differentiated cells that acquired a colonocyte/enterocyte-like phenotype). The culture medium was replaced every 2–3 days, with FBS lowered to 10% after cells had reached confluence. At day 0 or day 14, respectively, cells were washed once with PBS and scraped from the dishes, using HEPES-buffered saline (HBS; 50 mM HEPES, 150 mM NaCl, pH 7.4). Cells were then lysed by freezing/thawing and sonication, followed by centrifugation and determination of protein concentrations in the supernatants using the BCA protein assay kit (Thermo Fisher Scientific; Waltham, MA).

### 2.2 Construction of MGL and SELENBP1/MTO bacterial expression plasmids

Total RNA was isolated from differentiated human Caco-2 cells. Following reverse transcription, the coding sequence for human SELENBP1/MTO was amplified by PCR and ligated into the Twin-Strep-tag pASG-IBA102 vector (IBA Lifesciences, Göttingen, Germany; #5-4102-001) and subsequently into the DHFR control template vector (New England Biolabs, Ipswich, MA; #E6800S). For generation of SELENBP1 mutants (Gly225Trp, His329Tyr), site-directed *in vitro* mutagenesis was achieved by PCR using Phusion DNA Polymerase (Thermo Fisher Scientific) and subsequent digestion of the template strand with *Dpn*I (Thermo Fisher Scientific). The coding sequence for the *Brevibacterium aurantiacum* MGL [17], with an added 5′-terminal *Nde*I-site and a 3′-terminal Twin-Strep-tag and *Xho*I site was synthesized by ProteoGenix (Schiltigheim, France) and also ligated into the DHFR control template vector.

### 2.3 Bacterial overexpression and isolation of recombinant proteins

*E. coli* strains BL21 (New England Biolabs) and KRX (Promega, Madison, WI, USA) were transformed with the obtained plasmids. Bacteria were grown at 37 °C and 200 rpm in Luria/Miller LB medium (Carl Roth, Karlsruhe, Germany) supplemented with 3% ethanol and 50 μg/ml Carbenicillin (Carl Roth) until they reached an OD_600_ of 0.8. The temperature was then lowered to 22 °C, and biosynthesis of recombinant proteins was induced by adding 0.6 mM (final concentration) isopropyl β-d-1-thiogalactopyranoside (IPTG; Serva Electrophoresis, Heidelberg, Germany) for the BL21 or 0.06% (w/v) rhamnose (Carbolution Chemicals, St. Ingbert, Germany) for the KRX strain. After 3 h of incubation in the presence of the inducers, bacteria were centrifuged (6000×*g*; 4 °C; 5 min), and the pellets were frozen at −80 °C. For protein purification, bacteria were resuspended in HBS and lysed by sonication (58% output; 10 s pulse; 10 times). The soluble fraction was separated by centrifugation (4 °C; 16000×*g*; 15 min) and transferred to a Strep-Tactin XT gravity flow column (IBA Lifesciences). Protein purification was accomplished according to the manufacturer's protocol, with an additional washing step with 4 ml HBS containing 0.5% Tween 20 (Sigma-Aldrich). Alternatively, we washed three times using 1 ml HBS containing 20 mM MgCl_2_ (Carl Roth) and 5 mM ATP (Carl Roth) to elute contaminating proteins. Recombinant proteins were eluted using HBS containing 50 mM biotin (IBA Lifesciences). Buffer change of the protein solution was accomplished through ultrafiltration using a Vivaspin ultrafiltration column (cutoff: 10 kDa; Sartorius, Göttingen, Germany). Protein solutions were then aliquoted to avoid multiple freeze/thaw cycles and enzyme activity loss.

### 2.4 Methanethiol oxidase (MTO) assay: detection of the MTO reaction products H_2_S and H_2_O_2_

**Fig. 1:**
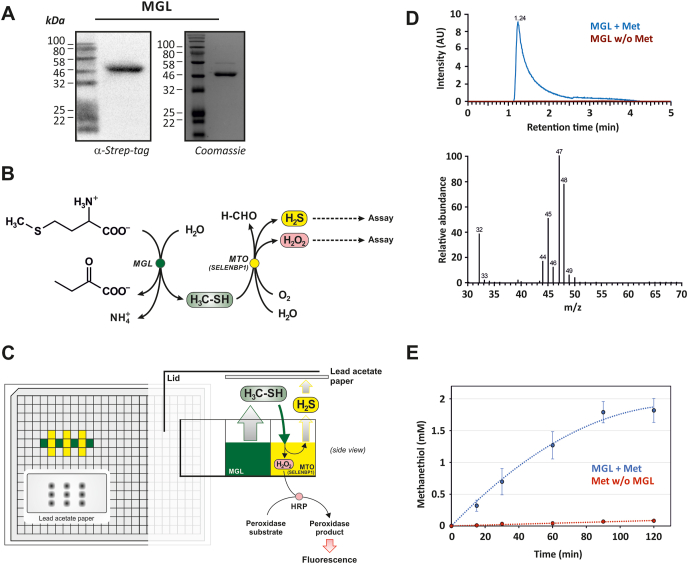
Coupled MTO assay, with *in situ*-generation of methanethiol through MGL-catalyzed methionine degradation. (A) MGL from *Brevibacterium aurantiacum* was epitope-tagged, expressed in *E. coli* and analyzed by immunoblotting (0.5 μg protein/lane) and staining of SDS-PAGE gels (2 μg protein/lane), respectively. (B) Reaction scheme of the coupled MTO assay. (C) Schematic layout of the MTO assay established here. (D) The MGL reaction releases methanethiol, as demonstrated by GC-MS-analysis. Extracted ion chromatogram (EIC) from the mass range 47.5–48.5, which corresponds to the molecular ion of methanethiol. Blue: reaction mixture containing MGL and Met (and PLP); red: reaction mixture without methionine. AU: arbitrary units. Bottom panel: corresponding mass spectrum of the MGL/Met reaction, which fits to the reported mass spectrum from the NIST-library (version 2016). (E) Methanethiol availability in MTO reaction wells, as assessed using DTNB. Blue: reaction mixture containing MGL and Met; red: reaction mixture without MGL. Data given as means ± SD of three independent measurements. (For interpretation of the references to color in this figure legend, the reader is referred to the Web version of this article.)

**Fig. 2:**
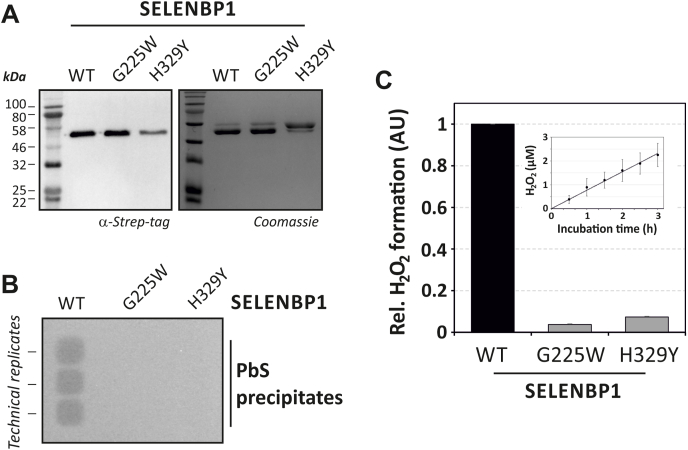
MTO activity of SELENBP1 and two variants found in individuals with extraoral halitosis. (A) SELENBP1 (wild-type, WT) as well as SELENBP1(Gly225Trp) and SELENBP1(His329Tyr) were epitope-tagged, expressed in *E. coli* and analyzed by immunoblotting (0.5 μg protein/lane) and SDS-PAGE (2 μg protein/lane), respectively. (B) Detection of H_2_S released during MTO reaction catalyzed by SELENBP1 (or its variants) as precipitates of PbS after interaction of H_2_S with lead (II) acetate. Data shown are representative of three independent experiments. (C) H_2_O_2_ released during 3 h of SELENBP1-catalyzed methanethiol oxidation as detected through HRP-catalyzed oxidation of a fluorescent probe. Values were normalized for content of the respective SELENBP1 variant and expressed as relative values, with data for WT-SELENBP1 set to 1. Inset: H_2_O_2_ concentrations increase linearly for at least 3 h during the assay. Data are given as means ± SD of three independent measurements (inset, 30 min: two measurements ± maximum deviation).

**Fig. 3:**
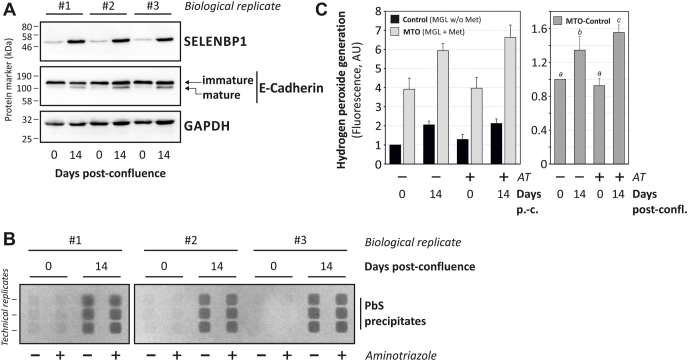
Both SELENBP1 levels and MTO activity increase in Caco-2 intestinal epithelial cells during differentiation. Caco-2 cells were cultured until reaching confluence (day 0) or until day 14 post confluence, followed by cell lysis. (A) SELENBP1, E-cadherin (as control for differentiation) and the housekeeping protein GAPDH (as control for equal loading) were detected in Caco-2 cell lysates by immunoblotting. (B, C) For the MTO assay, the soluble protein fraction of cell lysate (1 mg/ml) was incubated in the presence or absence of 1 mM of the catalase inhibitor 3-amino-1,2,4-triazole (AT). Samples were incubated with or without methionine (Met) as MGL substrate. (B) H_2_S was detected through precipitation as lead sulfide (PbS) upon interaction with lead (II) acetate. (C) H_2_O_2_ was quantitated in an enzymatic assay using a fluorescent probe, with data given as means +SD of three biological replicates. Values were normalized relative to the respective methionine negative control. Right panel: Data are differences between MTO and Control treatments, normalized to the treatment at day 0 without AT. Data are significantly different from each other if no labeling letter (a, b, or c) is shared between groups (p < 0.05 as determined by ANOVA, followed by Student-Newman-Keuls test).

MGL and MTO enzymatic reactions were set up in a 384-well plate (Greiner Bio-One, Frickenhausen, Germany; Fig. 1C). First, an MTO reaction well (yellow well in Fig. 1C) was prepared in a total volume of 40 μl/well, containing SELENBP1 or mutants thereof (at a final protein concentration of 0.225 mg/ml) or the soluble protein fraction of Caco-2 cell lysates (at a final protein concentration of 1 mg/ml) in HBS. Where indicated, the catalase inhibitor 3-amino-1,2,4-triazole (AT; Fluka/Sigma-Aldrich, #9540) was added to the Caco-2 lysates (1 mM final concentration). The MTO samples were applied as duplicates or triplicates in the vicinity of MGL wells, according to the scheme depicted in Fig. 1C. Thereafter, the MGL reaction was started by adding substrate solution (l-methionine in HBS) to MGL solution (MGL in HBS). In early experiments, the MGL cofactor pyridoxal phosphate (PLP, 1–2 mM; Sigma-Aldrich, #P9255) was added to the substrate solution. Later, it was left out as it made no difference; MGL was sufficiently saturated with PLP already after isolation. For experiments described in Fig. 2, 60 μl of substrate solution and 4.8 μl of MGL solution were combined, resulting in final concentrations of 0.154 mg/ml MGL and 20 mM methionine (Carl Roth, #1702.1) in HBS in a total volume of 64.8 μl. For experiments with Caco-2 lysates (Fig. 3), 13 μl of substrate solution and 27 μl of MGL solution were combined, resulting in final concentrations of 0.225 mg/ml MGL and 10 mM methionine in HBS in a total volume of 40 μl. The assay negative control was set up on a separate 384-well plate; here, the MGL reaction was initiated either without MGL or without methionine in the reaction mix. Empty wells adjacent to the reaction area were filled with 20 μl H_2_O.

After adding the MGL substrate solution, the plate was quickly covered by the lid, with Pb(II) acetate indicator paper for detection of H_2_S generation fixed underneath (see Fig. 1C), and sealed with adhesive tape. The indicator paper was prepared by soaking Whatman qualitative filter paper, grade 3 (Sigma-Aldrich, #1003-150) in 20 mM Pb(II) acetate (Sigma-Aldrich, #215902) for 30 min, followed by drying on a heating plate at 80 °C for 30 min [18]. The enzymatic reactions took place at 37 °C and under shaking at 200 rpm for 3 h (isolated SELENBP1 and variants) or 4 h (cell lysates). Thereafter, PbS spots on the indicator paper were detected with a ChemiDoc MP analyzer and Image Lab software (Bio-Rad Laboratories; Munich, Germany), and H_2_O_2_ generated during the MTO reaction was measured using the Fluorimetric Hydrogen Peroxide Assay Kit (# MAK165, Sigma Aldrich) at ʎ_ex_ = 540 nm and ʎ_em_ = 590 nm in a microplate reader (CLARIOstar; BMG Labtech; Ortenberg, Germany).

### 2.5 Detection of methanethiol using Ellman's reagent (DTNB)

To assess to what extent volatile methanethiol is available as MTO substrate when generated in an adjacent well of the 384-well plate, methanethiol-induced reduction of DTNB (5,5′-Dithiobis-2-nitrobenzoic acid; Carl Roth) to 2-nitro-5-thiobenzoate (TNB) was followed. The assay was prepared as described above, with 40 μl of DTNB solution (4 mg/ml DTNB in 0.1 M potassium phosphate buffer; 1 mM EDTA; pH 8.0) added instead of MTO samples in wells next to the MGL reaction well. In negative controls, the MGL reaction well contained methionine solution only, without added MGL. After starting the reaction (at 37 °C), aliquots were taken at different times, diluted 1:50 in potassium phosphate buffer (equilibrated to room temperature) and absorption was measured at 412 nm. Using the TNB extinction coefficient of 14,150 M^−1^ cm^−1^ [19], available methanethiol concentrations were assessed.

### 2.6 Detection of methanethiol by GC-MS

A 200 μl reaction mix containing 0.225 mg/ml of enriched MGL protein and MGL substrate solution (10 mM methionine, 1 mM PLP in HBS) was set up as described above. Incubation took place at 37 °C for 15 min in a 4 ml glass vial closed by a screw cap with a Teflon septum. Subsequently, a polydimethylsiloxane solid phase microextration (SPME) fiber (Supelco; Bellefonte, PA) was introduced into the headspace for 5 min at room temperature. The fiber was introduced into the hot (250 °C) injection port of the gas chromatograph in splitless mode. Analytes were separated with a Trace GC Ultra (Thermo Fisher Scientific) gas chromatograph equipped with a Zebron ZB-SemiVolatiles column (Phenomenex; Torrance, CA) with a linear temperature gradient (50–270 °C; 25 °C/min) and helium as carrier gas (1.2 ml/min). Detection of MT was carried out using an ISQ™ EC Single Quadrupole mass spectrometer (Thermo Fisher Scientific) with an electron impact (70eV) ionization source at 250 °C.

### 2.7 SDS-PAGE and immunoblot analysis

SDS-PAGE and immunoblotting were performed as previously described [12], with minor modifications. Cell lysates or enriched proteins were run on SDS-polyacrylamide gels and electroblotted onto PVDF membranes (Carl Roth). Proteins in the gels and on the membranes were stained with Coomassie Brilliant Blue (Sigma-Aldrich). The following mouse monoclonal antibodies were used for detection: anti-SELENBP1 (4D4; MBL Nagoya, Japan, #M061-3), anti-glyceraldehyde-3-phosphate dehydrogenase (GAPDH; GAPDH-71.1, Sigma-Aldrich, #G8795), anti-E-cadherin (67A4, Santa Cruz Biotechnology, Dallas, TX, USA, #sc-21791), anti-Strep-Tag II/StrepMAB (IBA Lifesciences), and horseradish peroxidase (HRP)-coupled anti-mouse IgG (Thermo Fisher Scientific). SuperSignal West Substrate (Thermo Fisher Scientific) was used for immunodetection on a ChemiDoc MP analyzer with Image Lab software (Bio-Rad Laboratories).

## 3 Results and discussion

### 3.1 Recombinant MGL as source of methanethiol, the substrate for a coupled MTO assay

To assess MTO activity of mammalian cells, we sought to provide substrate through enzymatic generation rather than via bulk application of methanethiol, a gas that is not trivial to handle. In line with microbial degradation of methionine being the major source of methanethiol in humans, we chose to generate methanethiol through a bacterial enzyme, MGL. To that end, we produced MGL from *Brevibacterium aurantiacum* [17] as a recombinant epitope-tagged protein with a predicted molecular mass of 43.8 kDa in *E. coli* (Fig. 1A). For analysis of MTO activity, methanethiol production was coupled to MTO-catalyzed oxidation, generating H_2_S, H_2_O_2_ and formaldehyde, followed by detection of H_2_S and H_2_O_2_ (Fig. 1B). Coupling of the two reactions was achieved through positioning the two reaction mixtures in separate, but adjacent wells of a 384-well plate (Fig. 1C): generated volatile methanethiol (green wells, Fig. 1C) diffuses to the neighboring well that contains the putative MTO (yellow wells, Fig. 1C). The 384-well plate is tightly sealed, but sufficient headspace is left above wells for diffusion of methanethiol. In order to adjust for potential inhomogeneity in methanethiol diffusion, a layout as described in Fig. 1C was chosen, with three technical replicates of the solution of the putative MTO positioned next to the MGL-containing well. A dry filter paper with lead acetate (PbAc_2_) was fixed on the bottom of the lid of the 384-well plate, covering the wells containing the samples. MTO-induced H_2_S release will result in black lead sulfide precipitates as indicated in Fig. 1C. H_2_O_2_ generated during MTO activity was detected from the same wells, but in a separate reaction: aliquots were taken from the wells containing the putative MTO and analyzed for the presence of H_2_O_2_ through peroxidase-catalyzed oxidation of a suitable probe.

To establish that methanethiol is generated in the MGL-catalyzed reaction, we tested for the release of methanethiol using GC-MS. A methanethiol-specific signal and mass fragmentation pattern resulted from analysis of the headspace above a solution containing MGL and methionine (Fig. 1D, blue line), but not in the absence of methionine (red line). Also, the formation of methanethiol was noticeable through its characteristic cabbage/cheese-like smell. To further demonstrate that methanethiol, once generated, also reaches the adjacent well (i. e. a yellow well in Fig. 1C), we tested for thiol-induced DTNB reduction in such a well, which, under the conditions employed here, in fact appears to occur in a linear fashion for at least an hour after starting the MGL reaction (Fig. 1E). Methanethiol concentrations achieved are in the millimolar range, likely allowing for sufficient saturation of SELENBP1, as the estimated K_m_-value of SELENBP1 for methanethiol is in the nanomolar range [5]. In summary, methanethiol is generated and is available as MTO substrate, using this experimental setting. Interestingly, supplementation of the MGL reaction mixture with the MGL cofactor, pyridoxal phosphate (PLP), did not have a significant effect on the results of the MTO assay (data not shown), suggesting that MGL isolated after bacterial overexpression was already sufficiently saturated with its cofactor.

### 3.2 Analysis of MTO activity of isolated wild-type and mutant human SELENBP1

We cloned and isolated bacterially expressed human SELENBP1 and generated two mutants thereof (Gly225Trp and His329Tyr) that were previously described in individuals suffering from familial extraoral halitosis [5]. Recombinant epitope-tagged proteins with a predicted molecular mass of 55.6 kDa were enriched as shown in Fig. 2A, with a purity of approximately 80–85% for wild-type SELENBP1. Impurities consisted of mainly one constituent that was identified by mass spectrometry as the 60 kDa bacterial chaperonin GroEL/Hsp 60. Interestingly, despite several attempts at enriching the His329Tyr variant of SELENBP1, we achieved a purity of only approx. 30%, which may imply decreased stability or proteolytic clearance of this mutant protein during heterologous expression.

Oxidation of methanethiol through MTO activity of SELENBP1 yields three products (Fig. 1B); two of these are being detected as part of the MTO assay presented here: H_2_S is trapped as black PbS that can be detected colorimetrically, and H_2_O_2_ is determined using a peroxidase-based fluorimetric assay.

Analysis of MTO activity of recombinant human SELENBP1 as well as the above-referenced mutants demonstrates that, while SELENBP1 is an active MTO, the Gly225Trp and His329Tyr variants are MTO-deficient versions of SELENBP1. H_2_S release (Fig. 2B) and H_2_O_2_ generation (Fig. 2C) were detected in SELENBP1-containing solutions, whereas the Gly225Trp variant yielded no detectable H_2_S or H_2_O_2_ production. The His329Tyr variant appeared to have some minute residual activity, as some H_2_O_2_ generation was detectable (Fig. 2C). The H_2_O_2_ production rate through MTO-catalyzed methanethiol oxidation is constant for up to 3 h (Fig. 2C, inset).

### 3.3 Enterocyte-like differentiation of Caco-2 cells is accompanied by parallel increases in SELENBP1 protein levels and MTO activity

Next, the established MTO assay was applied to test whether high SELENBP1 levels, previously reported for colonic enterocytes both in human colorectal tissue samples and in Caco-2 cells [13,14], come with elevated MTO activity. Confluent Caco-2 cells were held in culture for another 14 days, a procedure known to stimulate spontaneous enterocyte-like differentiation [20]. Maturation (truncation) of the cell adhesion protein E-cadherin occurs in Caco-2 cells [21], indicating differentiation [16]. Here, E-cadherin cleavage was also increased in differentiated cells at 14 days post-confluence compared to confluent cells (Fig. 3A). In parallel, SELENBP1 levels were upregulated about five-fold (densitometric analysis: 5.2 ± 1, means ± SD, n = 3; Fig. 3A), in line with previous proposals on SELENBP1 as a suitable marker of differentiation/maturation [[12], [13], [14]].

Analysis of MTO activities in the soluble fractions of Caco-2 cell lysates yielded a clear pattern: both H_2_S (Fig. 3B) and H_2_O_2_ (Fig. 3C) generation were significantly enhanced in lysates from differentiated (day 14 post-confluence), relative to those of undifferentiated (day 0) cells.

Lead sulfide precipitates resulting from hydrogen sulfide release using lysates of differentiated cells were strong and clearly discernible from control (i. e. without MGL added to the assay; not shown) and “undifferentiated” conditions (day 0, Fig. 3B). Low background signals were detectable also in undifferentiated cells, whereas negative controls (i. e. any samples without methionine added to the MGL reaction of the assay) were without discernible PbS precipitates (not shown). The weak PbS signals in undifferentiated cells likely reflect the low SELENBP1 levels found in these cells (Fig. 3A).

The generation of H_2_O_2_, the second MTO product tested for in our assay, was also enhanced in lysates of differentiated cells (Fig. 3C). Unlike H_2_S formation, increases in H_2_O_2_ generation were observed against a high background of basal H_2_O_2_ production in lysates also under control conditions (Fig. 3C). The overall increase of H_2_O_2_ release measured through the MTO assay was thus less extensive than the enhancement of H_2_S signals. This implies that both products should always be measured when performing an MTO assay, as sensitivity of the assay would be impaired to some extent, if only H_2_O_2_ were monitored: even under conditions causing a drastic difference in H_2_S release, the changes in H_2_O_2_ production were moderate.

Two factors are likely to cause the H_2_O_2_ response being moderate in the assay setup when analyzing MTO activity in cell lysates. First, the basal hydrogen peroxide generation appears to be slightly elevated in lysates of differentiated cells (Fig. 3C, black bars), implying that other peroxide-generating enzymatic systems are present in the lysates that are induced during differentiation. Second, basal hydrogen peroxide-reducing activities contribute to a dampened H_2_O_2_ signal in our assay: adding a catalase inhibitor, 3-aminotriazole (AT), to lysates prior to analysis of MTO activity significantly enhanced H_2_O_2_ signals (Fig. 3C), while leaving H_2_S generation unaltered (Fig. 3B). Addition of AT appears to increase assay sensitivity with respect to H_2_O_2_ formation: the differentiation-induced increase in MTO activity resulted in a 69% increase in H_2_O_2_ signal in the presence of AT, compared to only 35% in its absence (Fig. 3C, right panel).

Future investigations are warranted regarding a potential role of selenium and/or metal ions for the MTO activity of SELENBP1. Even though SELENBP1 was originally identified based on its capability to bind ^75^Se-labeled selenite [4], a more recent study found that it may also associate with zinc, copper and magnesium [22]. Moreover, binding of copper has been reported to be essential for the MTO activity of a bacterial SELENBP1 ortholog isolated from a *Hyphomicrobium* species [23]. Selenium, on the other hand, appears to bind to SELENBP1 mainly if applied at non-physiologically high doses [24]. Thus, SELENBP1 might provide an intracellular selenium buffer to cope with cytotoxic effects of selenite, as proposed for the *C. elegans* ortholog [25,26] rather than being dependent on selenium for its MTO activity. This idea is supported by a report showing SELENBP1 induction in human gastric cancer cells that were exposed to a cytotoxic dose of 30 μM selenite [27], whereas an adequate dose of 100 nM selenite did not result in elevated SELENBP1 levels in murine 3T3-L1 adipocytes [12]. Similarly, the SELENBP1 ortholog Y37A1B.5/SEMO-1 in the nematode *C. elegans* was increased only in response to high doses of selenite [26].

## 4 Conclusions

We here introduce a coupled enzyme assay for the detection of methanethiol oxidase activity. We applied this assay to recombinant human SELENBP1, demonstrating that two SELENBP1 mutants identified in individuals with extraoral halitosis, SELENBP1(Gly225Trp) and SELENBP1(His329Tyr) are MTO-inactive and of strongly decreased residual activity, respectively, in comparison to wild-type SELENBP1. Aside from a characteristic malodor, individuals harboring these altered SELENBP1 versions showed no apparent phenotypic alterations [5]. Similarly, Selenbp1-deficient mice also displayed no major phenotypical differences as compared to wild-type mice [5,28]. Gly-225 is evolutionarily conserved throughout all putative SELENBP1 orthologs, while His-329 is conserved only in eukaryotes [5,25].

**Fig. 4:**
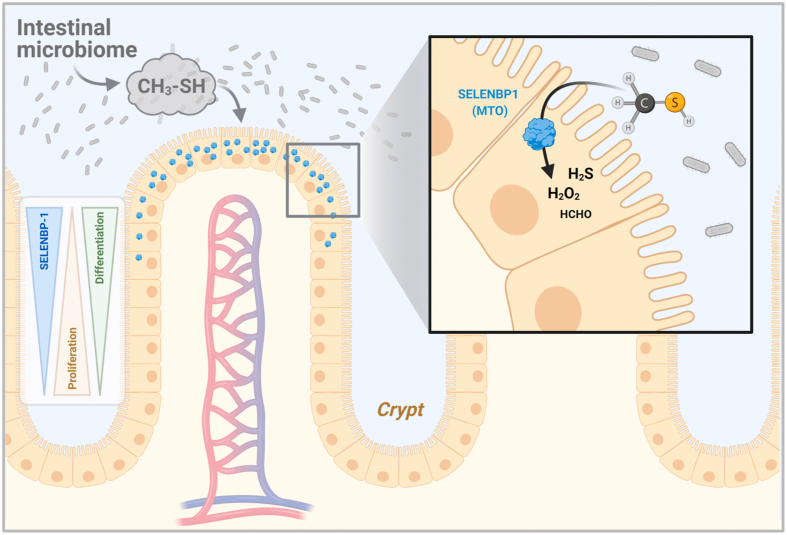
Schematic representation of the proposed role of SELENBP1 as a methanethiol oxidase (MTO) in the colon. SELENBP1 (blue protein symbol) expression in intestinal epithelial cells increases along with their differentiation. Highest SELENBP1 levels are found in the epithelial cells closest to the colonic lumen [13] and the microbiome, a major source of methanethiol. Upon diffusion into the epithelial cells, methanethiol is converted by SELENBP1 to hydrogen peroxide, hydrogen sulfide and formaldehyde. The reaction products, in turn, may affect cellular proliferation and differentiation by acting as redox signaling molecules. Scheme created with BioRender.com. (For interpretation of the references to color in this figure legend, the reader is referred to the Web version of this article.)

We also demonstrate that differentiation of cultured Caco-2 cells to an enterocyte/colonocyte-like phenotype comes with an enhanced specific MTO activity in parallel with SELENBP1 induction. Our assay imitates the *in vivo*-situation of SELENBP1 being located in direct proximity to the intestinal microbiome, a major source of methanethiol (Fig. 4): *in vivo*, a significant portion of methionine ingested as part of the diet is not absorbed within the small intestine and is then degraded by bacteria in the colon, resulting in methanethiol release; *Citrobacter freundii*, *Morganella morganii* and several *Proteus* species were described as being among the intestinal bacteria contributing to this methanethiol generation [29].

The upregulation of SELENBP1 during cell differentiation along the crypt-lumen axis, which entails high SELENBP1 levels in cells at the upper end of the crypts close to the colonic lumen (Fig. 4), suggests that intestinal SELENBP1 serves the purpose of coping with bacteria-derived methanethiol. It is, however, unclear whether this is to be understood as a protective mechanism against this toxic volatile molecule, as neither humans nor mice with SELENBP1 deficiency do appear to suffer from gastrointestinal issues [5,28]. An alternate view would focus on the products of the MTO reaction. In this regard, both H_2_O_2_ and H_2_S are redox signaling molecules, affecting regulatory pathways implicated in cell proliferation and differentiation, such as in adipocytes and colonic enterocytes [12,[30], [31], [32]]. SELENBP1/MTO could therefore be a cellular source of these signaling molecules in some tissues. In line with this notion, SELENBP1-generated H_2_S was recently shown to promote adipogenesis and lipid accumulation in adipocytes [33]. On the other hand, decreases in SELENBP1 levels have been reported for many types of cancer, including colorectal carcinomas. It has been hypothesized that down-regulation of SELENBP1 may contribute to the aberrant proliferation of tumor cells [13,34]. Given the close association between SELENBP1 expression and MTO activity demonstrated here, our MTO assay may allow for a more rapid, simple and quantifiable determination of SELENBP1 levels in tumor tissue than the conventionally used methods of immunoblotting and immunohistochemistry. This type of screening for SELENBP1/MTO may be of interest as low SELENBP1 levels in tumor tissue are associated with poor prognosis for patients [13,34].

## Declaration of competing interest

The authors have no conflict of interest to declare.
